# 

**Published:** 1887-04

**Authors:** 


### CONTENTS.

COMMUNICATIONS.

Irregularities.......... 161
A Ten Minutes Sermon on Nutrition.......... 169
The Relation of Dentistry and Medicine.......... 175
New Remedies.......... 179
Mississippi Valley Association of Dental Surgeons.......... 181
Minutes of Mississippi Valley Association of Dental Surgeons.......... 192

LEGISLATION.

An Act to Regulate the Practice of Dentistry in Alabama.......... 199
The Dental Law of Indiana as Amended in 1887.......... 201

SELECTIONS.

Surgical Aphorisms.......... 204
A New Method for the Restoration of Respiration Lost under Chloroform.......... 207
Lister's Latest Antiseptic.......... 208
SALMAGUNDI.......... 210

EDITORIAL.

A Well Deserved Honor.......... 212

### THE DENTAL REGISTER,

A MONTHLY MAGAZINE DEVOTED TO THE INTERESTS OF THE

**DENTAL PROFESSION.**

*Terms Reduced to* $2.00 *Per Year.* *Single Copies,* 25 *Cents.*

**EDITED BY PROF. J. TAFT, D.D.S.**

—AND—

**Published by SAM'L A. CROCKER & CO., Ohio Dental and Surgical Depot.**

The volume commences in January and closes in December.

The REGISTER being issued monthly is always fresh, therefore Indispensable to the practitioner who desires to keep posted up with the new inventions and improvements which are daily developed. Neither expense nor effort will be spared to give value and variety to its contents. Articles will be illustrated with well executed engravings whenever they will add to the interest or assist to explanation.

Its extensive circulation and frequency of issue make it one of the BEST DENTAL ADVERTISING MEDIUMS in the United States.

All subscriptions must begin with January or July.

Local or special notices, five lines or less, will be inserted for $3 each insertion; over five lines, 50 cts. per line.

All advertising bills payable in advance, or at furthest, after the issue of the first number containing the advertisement. All transient advertisements to be paid for in advance.

☞CONTRIBUTIONS SOLICITED.

Exclusively Editorial Communications should be addressed to the Editor.

The latest number of the REGISTER may be ordered with or without other goods at any time, and all letters should be addressed to

SAM'L A. CROCKER & CO., Ohio Dental and Surgical Depot, Cincinnati, O.

